# Cubitus varus deformity following paediatric supracondylar humeral fracture remodelling predominantly in the sagittal direction: A three-dimensional analysis of eighty-six cases

**DOI:** 10.1007/s00264-024-06197-2

**Published:** 2024-05-10

**Authors:** Tasuku Miyake, Satoshi Miyamura, Ryo Miki, Ryoya Shiode, Toru Iwahashi, Arisa Kazui, Natsuki Yamamoto, Hiroyuki Tanaka, Seiji Okada, Tsuyoshi Murase, Kunihiro Oka

**Affiliations:** 1 Department of Orthopedic Surgery, Osaka University Graduate School of Medicine, Suita, Japan; 2 Miki Orthopedic Surgery & Internal Medicine, Minoh, Japan; 3 Department of Sports Medical Science, Osaka University Graduate School of Medicine, Suita, Japan; 4 Department of Orthopedic Surgery, Bell Land General Hospital, Sakai, Japan; 5 Department of Orthopedic Biomaterial Science, Osaka University Graduate School of Medicine, Suita, Japan

**Keywords:** Cubitus varus deformity, Three-dimensional analysis, Remodelling, Complication after supracondylar fracture, Elbow

## Abstract

**Purpose:**

Three-dimensional (3D) capacity for remodelling in cubitus varus deformity (CVD) after paediatric supracondylar humeral fractures (PSHFs) remains unelucidated. This study investigated remodelling patterns after PSHFs by examining 3D deformity distribution over time after injury.

**Methods:**

Computed tomography (CT) data of 86 patients with CVD after PSHFs were analysed. The 3D deformity angles in the sagittal, coronal, and axial directions were assessed and correlated with the duration between the age at injury and CT evaluation. For the subgroup analysis, we performed the same correlation analysis in a younger (< 8 years old) and an older group (≥ 8 years old); we categorized the duration into early (< 2 years), middle (≥ 2 to < 5 years), and late periods (≥ 5 years) and compared the deformity angles of each direction among the three groups.

**Results:**

Sagittal deformity showed a moderate correlation with the duration of deformity (r = -0.54; P < 0.001), while coronal and axial deformities showed a negligible correlation. Sagittal deformity showed moderate correlations with the duration in the younger group (r = -0.62; P < 0.001) and weak correlations in the older group (r = -0.37; P = 0.091). In the sagittal direction, the deformity angle in the early period was significantly larger than those in the mid and late periods (P < 0.001). However, there were no significant differences among the three groups in the coronal and axial directions.

**Conclusion:**

Sagittal deformities in CVDs are capable of remodelling, especially in the early period and at a younger age, whereas coronal and axial deformities are less likely to undergo remodelling.

**Supplementary Information:**

The online version contains supplementary material available at 10.1007/s00264-024-06197-2.

## Introduction

Owing to inadequate treatment, paediatric supracondylar humeral fractures (PSHFs) often heal with deformities, most notably, cubitus varus deformity (CVD), in 10%–57% of cases [1–3]. The residual combined deformity comprises varus, extension, and rotation [3, 4], with unacceptable cosmetic defects that also induce functional effects, such as restricted motion at the elbow joint, recurrent fractures of the lateral humeral condyle [5], posterolateral rotatory instability [6], late ulnar nerve neuropathy [7, 8], and osteoarthritis (OA) [9, 10].

Nonetheless, fractures in children have a good capacity for remodelling. Although remodelling of sagittal angulation generally occurs [11–13], remodelling of the coronal and rotational malunion is not expected [12]. However, some sceptical reports exist regarding the capacity for remodelling in the sagittal direction after PSHFs [14, 15].

Most reports on remodelling relied on a two-dimensional analysis using conventional radiographic measurements [11, 12, 16–18], which when compared with three-dimensional (3D) evaluation using computed tomography (CT), can generate an error of 10° or more in a CVD [3]. Furthermore, evaluating the residual rotational deformity on anteroposterior and lateral radiographs is challenging [3, 19, 20]. Although CVD remodelling should be evaluated three-dimensionally with CT, the 3D capacity of remodelling is unelucidated.

We hypothesized that CVDs would exhibit a unique remodelling pattern. We aimed to investigate the remodelling patterns following PSHFs by examining the distribution of 3D deformities over time after injury.

## Materials and methods

### Study Design

Ninety-two consecutive patients with CVD, who visited our institution between September 2005 and December 2022, underwent CT scanning of both elbows for an evaluation of the deformity or a preoperative simulation. This study was approved by our institutional review board. The requirement for written informed consent was waived because of the retrospective nature of the study.

The inclusion criterion was the presence of a CVD compared to the unaffected side, resulting from the malunion of a supracondylar fracture of the distal humerus. Six patients were excluded: four with elbow OA, one with bilateral deformities, and one with a congenital deformity. The remaining 86 patients (71 males, 15 females) were enrolled (Table 1). For remodelling evaluation, we calculated the age as 20 years if the patient was ≥20 years old at the time of CT evaluation because fracture remodelling is not expected after 20 years of age [21]. In the 12 patients aged ≥20 years, we confirmed on the plain radiographs that the epiphyseal line was closed.

**Table 1:** Characteristics of the study cohort

	Patients (n = 86)
Sex	71 males, 15 females
Affected side	Right, 43; Left, 43
Age at injury (years)	5.7 years (± 2.6 years, 1–12)
Age at time of CT Scan (years)	11.4 years (± 4.7 years, 4–20)
Duration of deformity (years)	5.8 years (± 4.4 years, 0–17)
Initial treatment	Casting; 40 (47%) Pinning; 31 (36%) Unknown; 15 (17%)

CT, computed tomography

### Image Acquisition and 3D Bone Model Reconstruction

We used a helical-type CT scanner (LightSpeed Ultra 16 or 64; GE Healthcare, Waukesha, Wisconsin) with a low radiation-dose technique (slice thickness, 1.25 mm; pixel size, 0.75–0.85 mm; scan time, 0.5 s; scan pitch, 0.562:1; tube current, 20–150 mA and tube voltage, 120 kV) [3, 10, 22] to evaluate the 3D deformity. With the patient in the prone position, both the upper limbs were scanned with the shoulder at full elevation, the elbow at full extension, and the forearm maintained in supination. The digital data were saved and computer analysed. We created 3D surface models of the bilateral humerus, radius, and ulna from the digital data by semiautomatically segmenting individual osseous regions using a global threshold algorithm, with a threshold of 250 Hounsfield units (HU) [23] using MvIndex/BoneSimulator image processing software (Teijin Nakashima Medical, Okayama, Japan).

### Quantification of Deformity

**Fig. 1:**
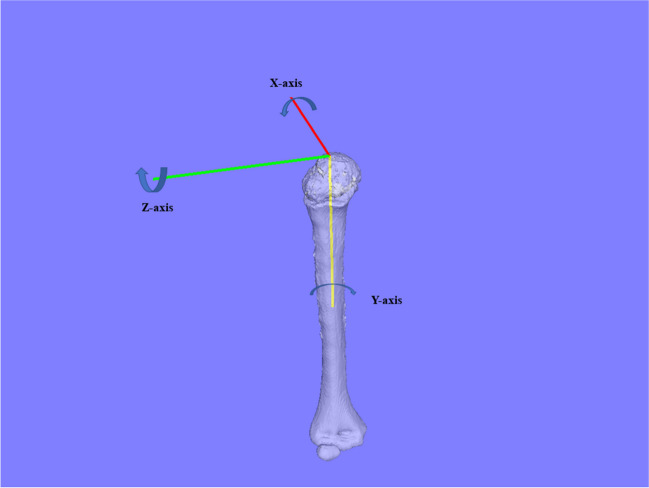
Orthogonal coordinates of distal humerus. The coordinate system was a modified version of the International Society of Biomechanics reference system. The origin is the intersection of the humeral inertia axis (y-axis) and the proximal end of the humeral head. Z, Line connecting the origin and the parallel line connecting the lateral and medial epicondyle tips of the humerus; X, line perpendicular to the YZ plane

To quantify deformities of the distal humerus, we used a modified orthogonal reference system originally advocated by the International Society of Biomechanics [24]. The origin was defined as the intersection of the humeral inertia axis (Y-axis) and the proximal end of the humeral head, and was indicated as distal ( +) or proximal (-). The Z-axis was defined as the line connecting the origin and the parallel line connecting the lateral and medial epicondyle tips of the humerus that indicated the lateral ( +) or medial (-) direction. The X-axis was defined as the line perpendicular to the YZ plane and indicated the posterior ( +) or anterior (-) direction (Fig. 1).

**Fig. 2:**
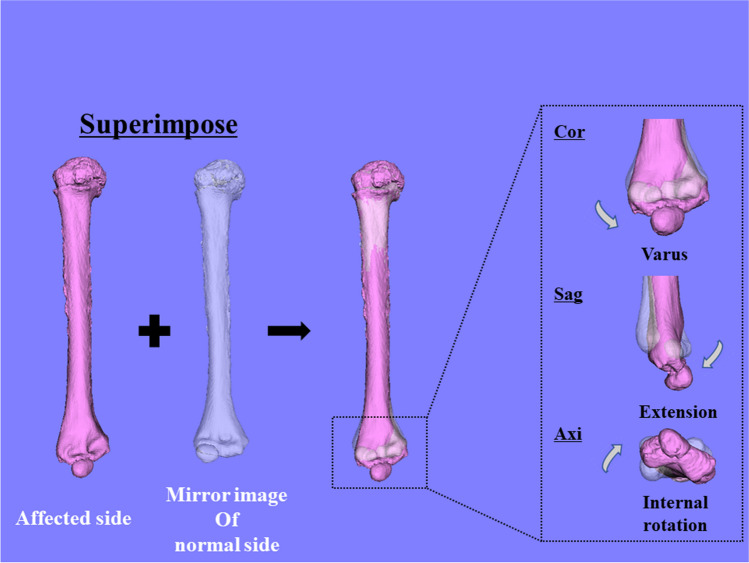
Three-dimensional (3D) deformity of the distal humerus The proximal site of the affected humerus is superimposed on a mirror image of the contralateral normal humerus. Next, the 3D deformity of the distal site was quantified in three directions: varus (coronal direction), extension (sagittal direction), and rotation (axial direction)

To evaluate the 3D humerus deformity using a surface-based registration technique [25, 26], the affected humerus was compared with a mirror image of the contralateral normal humerus (Fig. 2). The 3D varus, extension, and internal rotation deformities were quantified by superimposing the proximal parts of the affected humerus onto those of the mirror image of the normal humerus and calculating the rotation angles of the distal parts, according to previous studies that used the Euler angle method [3, 10]. In this study, we calculated the absolute values of the deformity angles in the coronal, sagittal, and axial directions relative to those in the contralateral normal humerus to investigate the remodelling. In our cohort, 43 (50%), 19 (22%), 14 (16%), and ten (12%) participants had coronal–axial, triplane, coronal, and coronal–sagittal direction deformities, respectively; a deformity > 5° was considered significant [27, 28].

### Statistical analysis

All statistical analyses were performed using GraphPad Prism version 9.0 (San Diego, CA, USA); the significance level was set at a *P*-value < 0.05.

In general, supracondylar fractures of the humerus occur predominantly at four to seven years of age [16, 29], with a male-to-female ratio of 3:1 [30]. Only a varus deformity is seen in 20% of patients, whereas the remaining 80% have a combined deformity [3]. In the current cohort, the mean age at injury was 5.7 years, with a male-to-female ratio of 4.7:1; 14 cases (16%) showed only varus deformity. Therefore, we performed a correlation analysis to evaluate the trend in deformities with the assumption, based on previous reports, that the study population was a generalized population. This study did not follow changes in each patient over time, but instead made fixed-point observations within the population to examine correlations. Spearman’s correlation coefficients were determined between the 3-D deformity angle in each direction (sagittal, coronal, and axial) and the duration of deformity and between the 3D deformity and age at injury. The strength of correlation was classified as negligible (|r|< 0.2), low (|r|= 0.2–0.4), moderate (|r|= 0.4–0.7), or high (|r|> 0.7).

Subsequently, to examine the timing of remodelling, we divided the patients into three groups based on the duration between ages at injury and CT evaluation: an early-period (< 2 years), mid-period (≥ 2 to < 5 years), and a late-period (≥ 5 years) [31]. The 3D deformity angles were analysed using the Kruskal–Wallis nonparametric analysis followed by Dunn’s multiple-comparison test to evaluate differences among the three periods in each direction.

Furthermore, using Spearman’s correlation coefficient, we correlated the 3D deformity angles with the duration at injury in a younger group (< 8 years) and an older group (≥ 8 years) to investigate the impact of age at injury on remodelling. A threshold age of 8 years was determined, considering that children older than 8 years have minimal capacity to remodel sagittal plane malunion [11].

Power analysis performed to calculate the sample size necessary to detect the correlation (calculated with effect size = 0.4, alpha = 0.05, two-tailed, power = 0.95) indicated that a sample size of 71 participants was sufficient.

## Results

In the study cohort, the right and left sides were affected in 43 patients each; the mean age at injury was 5.7 years (range, 1–12 years), and the mean age at the time of CT image acquisition was 11.4 years (range, 4–20 years). The mean interval between the original injury and image acquisition was 5.8 years (range, 0–17 years).

### 3D Humerus Deformity

The mean deformity angles in sagittal, coronal, and axial directions were 5.9° ± 8.4° of extension, 16.8° ± 5.6° of varus, and 11.8° ± 9.4° of internal rotation, respectively.

### Correlation Analysis Between 3D-deformity Angles and Duration of Deformity

**Fig. 3:**
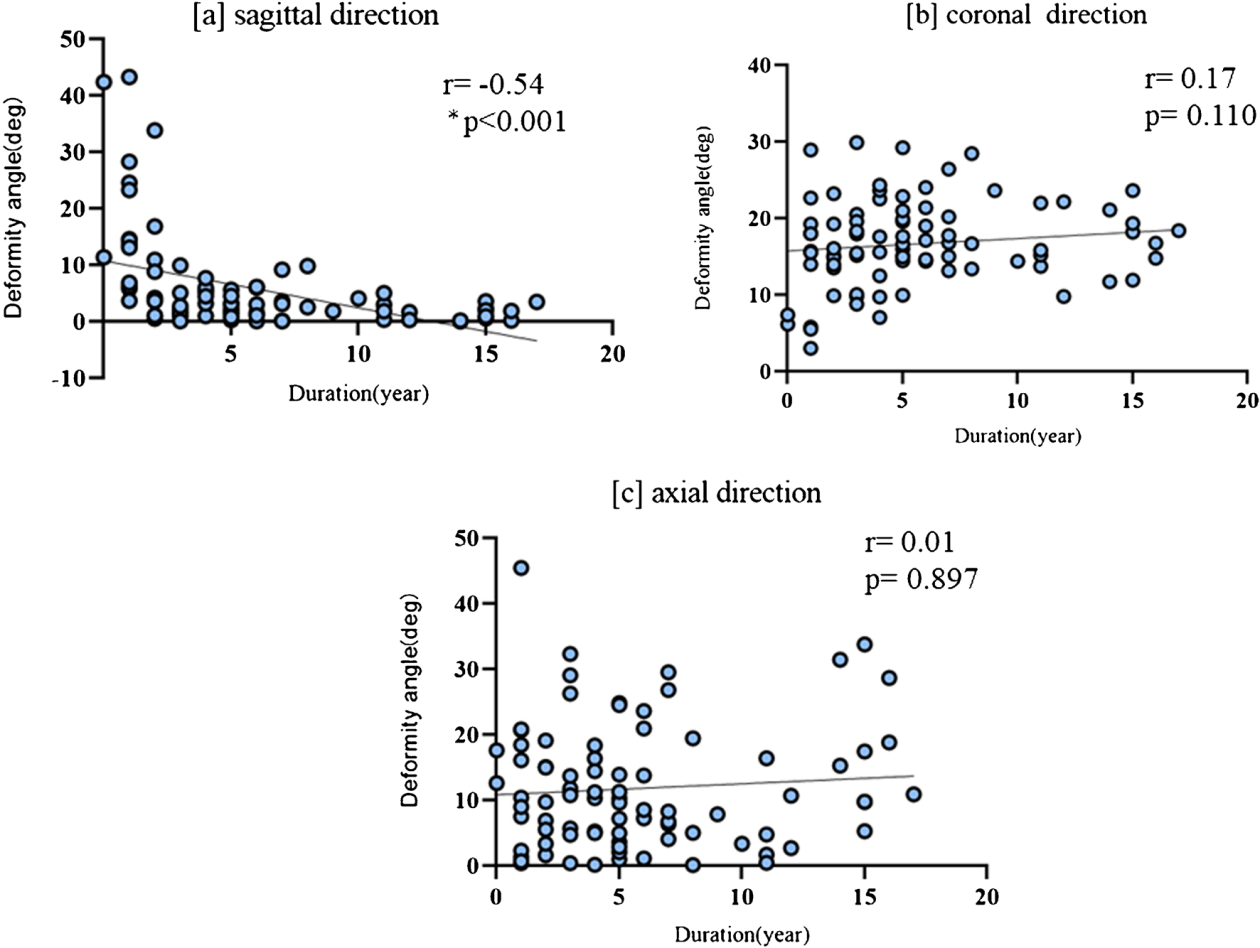
The graph shows the correlation between 3D deformity angles and duration with deformity at each direction (**a**) Sagittal direction deformity; (**b**) Coronal direction deformity; and [c] Axial direction deformity. Only in the sagittal direction was a negative moderate correlation between them observed. SD, Three-dimensional

There was a moderate negative correlation between the sagittal direction deformity and the duration of deformity (r =  − 0.54; *P* < 0.001). In contrast, coronal (r = 0.17; *P* = 0.110) and axial (r = 0.01; *P* = 0.897) deformities showed negligible correlations with the duration of deformity (Fig. 3).

### Comparison of 3D-deformity Angles Among Early, Middle, and Late Periods in Each Deformity Direction

**Fig. 4:**
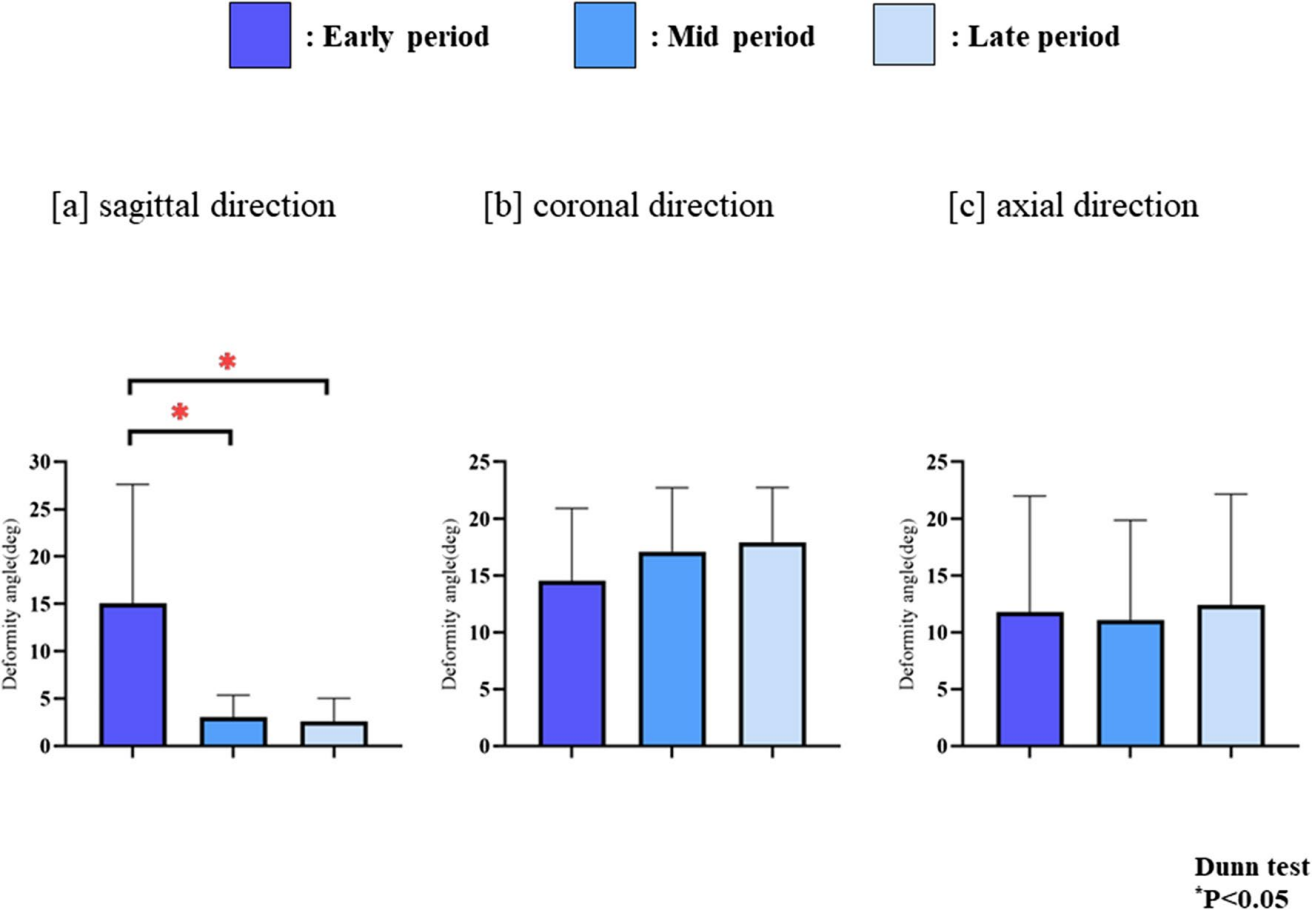
Deformity angles In the sagittal direction deformity, the middle (n = 31) and late-stage (n = 33) groups showed significantly reduced deformity angles compared to those in the early-stage group (n = 22) (Kruskal–Wallis/Dunn’s, *P* < 0.05)

In the sagittal direction deformity, the deformity angle of the early-period (15.1° ± 12.6°) was significantly higher than that of the middle (3.1° ± 2.3°, *P* < 0.001) and late-periods (2.6° ± 2.5°, *P* < 0.001); however, there was no significant difference between the deformity angles of the middle and late-periods (*P* = 1.000). In the coronal direction deformity, there was no significant difference among the deformity angles of early (14.5° ± 6.4°), middle (17.1° ± 5.6°), and late (18.9° ± 4.8°) periods (early vs. middle, *P* = 0.386; early vs. late, *P* = 0.173; middle vs. late, *P* = 1.000). Similarly, in the axial direction deformity, there was no significant difference among the deformity angles of early (11.8° ± 10.2°), middle (11.1° ± 8.8°), and late (12.4° ± 9.8°) periods (early vs. middle, *P* = 1.000; early vs. late, *P* = 1.000; and middle vs. late, *P* = 1.000); Fig. 4).

### Correlation Analysis Between Deformity and Deformity Duration in the Younger and Older Groups

**Fig. 5:**
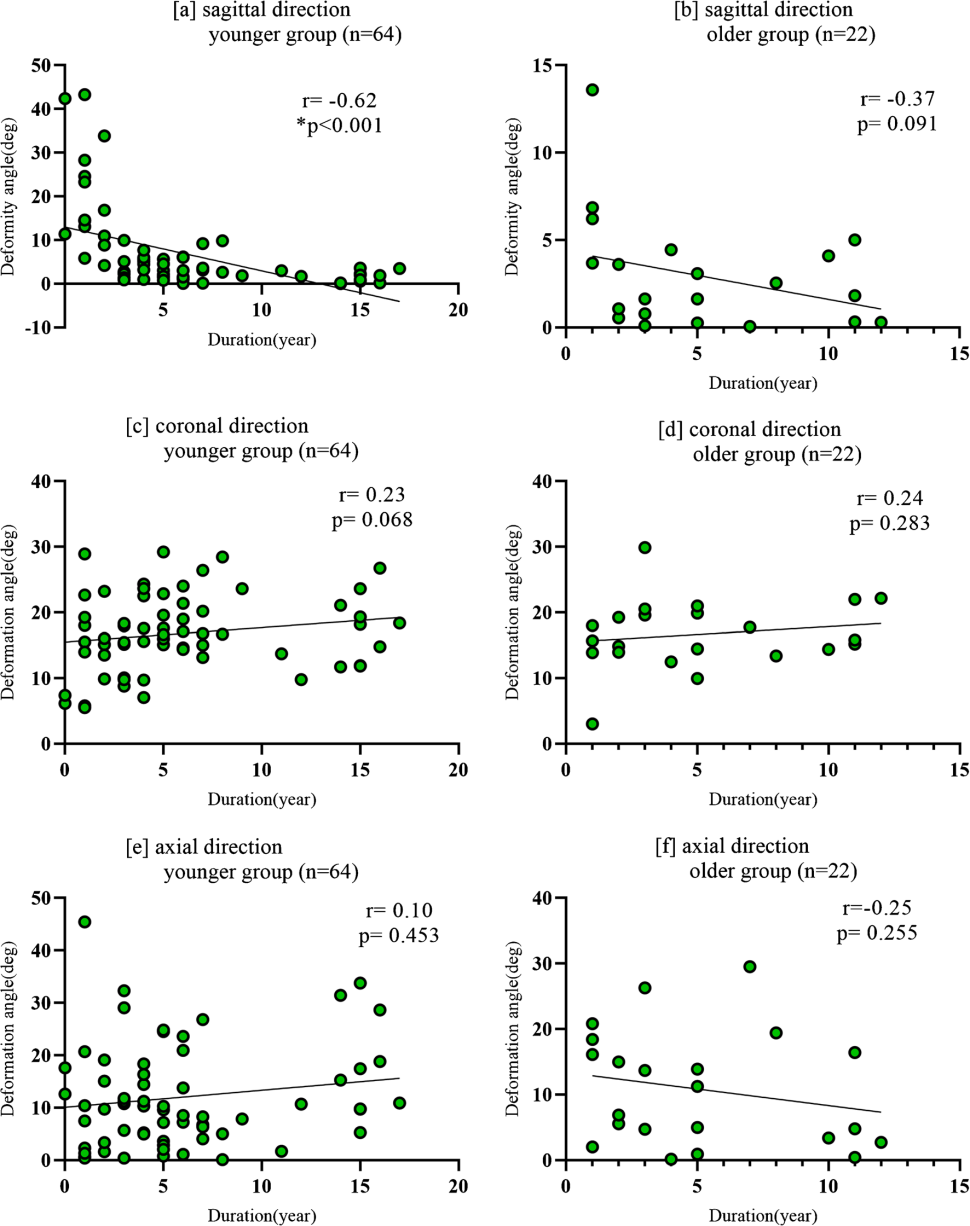
The graph shows the correlation between the deformity angle and the duration with deformity at each direction in the younger group (**a**, **c**, **e**) and the older group (**b**, **d**, **f**) The correlation between the deformity angle in the sagittal direction and the duration was moderately negative in the younger group (**a**); however, no significant correlation was observed in the older group (b). No significant correlation was found in the coronal or axial direction between the two groups (**c**–**f**)

There was a moderate negative correlation between the sagittal direction deformity and the duration of deformity in the younger group (r =  − 0.62; *P* < 0.001), whereas there was a weak correlation between these in the older group (r =  − 0.37; *P* = 0.091). Coronal-direction deformities showed a weak correlation with duration in the younger group (r = 0.23; *P* = 0.068) and the older group (r = 0.24; *P* = 0.283). Axial-direction deformities showed a negligible correlation with duration in the younger group (r = 0.10; *P* = 0.453) and a low negative correlation in the older group (r =  − 0.25; *P* = 0.255; Fig. 5).

## Discussion

We analysed the 3D capacity for remodelling after supracondylar humeral fracture (SHF) by investigating the distribution of deformity angles among 86 CVDs with varying periods since injury. Our results indicate that sagittal deformities are capable of remodelling, whereas coronal and axial deformities are less likely to undergo remodelling. A subgroup analysis revealed that the sagittal direction undergoes robust remodelling in the early period following injury, particularly in younger age groups.

Several studies have analysed CVD remodelling using conventional radiography, showing that deformities in the sagittal direction can be remodelled. Persiani et al. [15] analysed 62 children with extension-type SHFs with a mean follow-up of four years and three months. Guven et al. [32] analysed data from 49 children with Gartland type 3 fractures with a mean follow-up of 22.4 years. Conventional radiographic measurements were obtained preoperatively and at the final follow-up to examine the remodelling in each direction; a greater capacity for remodelling was observed in the sagittal direction compared to that in the coronal direction. James et al. [11] reported 100% remodelling in the sagittal direction in 41 children under 5 years. However, many investigators proposed that deformity remodelling in the coronal direction is unlikely [16, 33–36], which has been consistently observed. Remodelling in the axial direction is as unlikely as in the coronal direction [37]; however, no studies have accurately evaluated remodelling in the axial direction using 3D images. The sagittal deformity angles showed a moderate negative correlation with the duration of the deformity when considering the time interval from injury. This indicates that sagittal deformities are capable of remodelling.

Fractures in children are typically remodelled according to Wolff’s law [38]: new bone is laid down on the compressed or concave side of the long bone. Moreover, remodelling is more likely to occur spontaneously if the child is younger, if the fracture site is closer to the physis, or if there is a relative alignment of angulation in the normal plane of motion of the joint [37]. Our results showed that deformities in the sagittal direction were predominantly remodelled. Presumably, the direction of the sagittal deformity coincided with the direction of the flexion–extension motion of the elbow joint, which may have influenced the deformity remodelling in the sagittal direction. During elbow flexion, the flexor muscles overpower the extensor muscles, generating compressive forces on the flexor side of the fracture and traction forces on the opposite side. This results in active bone formation on the flexor side and bone resorption on the extensor side [37].

Although no previous report exists on the timing of remodelling after SHFs, distal radius fractures in children mainly remodel in the first year after injury and are less likely to remodel after ≥two 2 years [31, 39]. We found that sagittal deformity in the early period was significantly greater than that in the middle and late periods when classified into three groups, based on the duration between age at injury and CT evaluation. This indicates that the sagittal deformity of the distal humerus was remodelled vigorously in the early period after SHFs, and.

in the younger group showed a moderate correlation with duration. In general, the younger the age at injury, the greater the ability to remodel. One possible explanation is that the periosteum and endosteum play important roles in bone remodelling, and these tissues are more active in the younger group, making remodelling more likely to occur [37]. Camus et al. [40] reported that children older than nine years may have a minimal capacity for remodelling, which supports the current findings that remodelling ability decreases with age with a threshold of eight years. In later life, posterolateral rotatory instability [6], late ulnar nerve neuropathy [7, 8], and osteoarthritis [9, 10] are known late complications of CVD, and these may be somehow related to the specific remodelling patterns identified in this study.

Collectively, residual deformities after SHFs can be remodelled in the sagittal direction, and sagittal remodelling is more likely to occur early after injury and at younger ages. These findings are useful for optimizing treatment strategies and evaluating the prognosis of fractures clinically. First, it is important to pay particular attention to the residual deformity in the coronal and axial directions at the initial treatment and during the follow-up period when treated conservatively or surgically. Second, if these deformities remain cosmetically and functionally problematic, corrective osteotomy is necessary at any age to prevent these late complications because the deformities in the coronal and axial direction will not be remodelled. However, if the deformity in the sagittal direction is the predominant deformity, there is a possibility of remodelling, and the patients younger than 8 years should be followed up. Corrective osteotomy is then decided when there is functional impairment such as limited range of motion and remodelling is not expected.

The current study has some limitations. First, this study investigated the distribution of deformities in all age groups, and individual cases were not followed up. However, we believe that remodelling can be evaluated by investigating the distribution because we extracted data from the general population after SHFs. Second, patient information was limited to medical records and X-ray/CT images because this was a retrospective study.

In conclusion, with CVD following PSHF, there was a predominant correlation between sagittal deformity and duration. Sagittal deformities are capable of remodelling, whereas coronal and axial deformities are less likely to undergo remodelling.

We believe that our findings will improve the understanding of the remodelling potential of CVDs. Furthermore, the insight presented herein may contribute to the determination of treatment strategies for SHFs and CVDs.

### Supplementary Information

Below is the link to the electronic supplementary material.

Supplementary file1 (DOCX 406 KB)

Supplementary file2 (DOCX 28 KB)

Supplementary file3 (DOCX 22 KB)
