# Supplementary material for: Cubitus varus deformity following paediatric supracondylar humeral fracture remodelling predominantly in the sagittal direction: A three-dimensional analysis of eighty-six cases
Source: Int Orthop. 2024 May 10;48(8):2091–9. doi: 10.1007/s00264-024-06197-2 (PMC11246304; doi:10.1007/s00264-024-06197-2)
Supplement: Supplementary file 1 — Supplementary file1 (DOCX 406 KB) [file 264_2024_6197_MOESM1_ESM.docx]

**Journal name: *International Orthopaedics***

**Title: Cubitus varus deformity following paediatric supracondylar humeral fracture remodelling predominantly in the sagittal direction: A three-dimensional analysis of eighty-six cases**

**Running Title: Remodelling capacity of cubitus varus deformity**

Tasuku Miyake, MD^a^ , Satoshi Miyamura, MD, PhD^a^ , Ryo Miki, MD^b^ , Ryoya Shiode, MD, PhD^a^ , Toru Iwahashi, MD, PhD^a^ , Arisa Kazui, MD^a^ , Natsuki Yamamoto, MD^a^ , Hiroyuki Tanaka, MD, PhD^a, c^ , Seiji Okada, MD, PhD^a^, Tsuyoshi Murase, MD, PhD^a, d^, Kunihiro Oka, MD, PhD^a, e^

^a^ Department of Orthopedic Surgery, Osaka University Graduate School of Medicine, Suita, Japan

^b^ Miki orthopedic surgery & internal medicine, Minoh, Japan

^c^ Department of Sports Medical Science, Osaka University Graduate School of Medicine, Suita, Japan

^d^ Department of Orthopedic Surgery, Bell Land General Hospital, Sakai, Japan

^e^ Department of Orthopedic Biomaterial Science, Osaka University Graduate School of Medicine, Suita, Japan

**Corresponding author:** Kunihiro Oka, MD, PhD

**Postal Address:** Department of Orthopedic Biomaterial Science, Osaka University Graduate School of Medicine, 2-2 Yamadaoka, Suita, Osaka 565-0871, Japan

**Email address:** [oka-kunihiro@ort.med.osaka-u.ac.jp](mailto:oka-kunihiro@ort.med.osaka-u.ac.jp)

**Phone:** +81-6-6879-3552; **Fax:** +81-6-6879-3559

**ORCID:**

Tasuku Miyake: 0009-0001-7404-2460

Kunihiro Oka: 0000-0002-7770-4634

**Radiographic comparisons between original injury and CT scans**

We analysed the radiographic comparisons between original injury and CT scans in 17 patients in as many cases as possible because there were no radiographic data available for all patients at bone union. The 17 patients (10 males, 7 females) were enrolled (Supplementary Table 1). Patient data showed a similar trend in all patients in this study. Based on previous reports [3], we measured the humerus-elbow-wrist angle (HEW-A) on an anteroposterior radiographic view of the elbow and the tilting angle (TA) on a lateral radiographic view of the elbow at bone union and CT scans (Supplementary Fig. 1). The mean HEW-A at bone union and at CT evaluation were -13.5°±7.3° and -12.6°±6.6°, respectively, and the mean TA at bone union and at CT evaluation were 19.8°±13.3° and 31.6°±14.9°, respectively. For date analysis, paired t-tests were performed for within HEW-A group and TA group comparisons. The HEW-A group showed no significant change (*P* =0.051), while the TA group showed significant improvements (*P* < 0.001) (Supplementary Fig. 2). This result showed that the deformity in the coronal direction remained almost unchanged, while the deformity in the sagittal direction showed a tendency to remodel. These results supported our findings.

**Supplementary Table 1.** Patient data.

CT, computed tomography; SD, standard deviation; TA, tilting angle; HEW-A, humerus-elbow-wrist angle


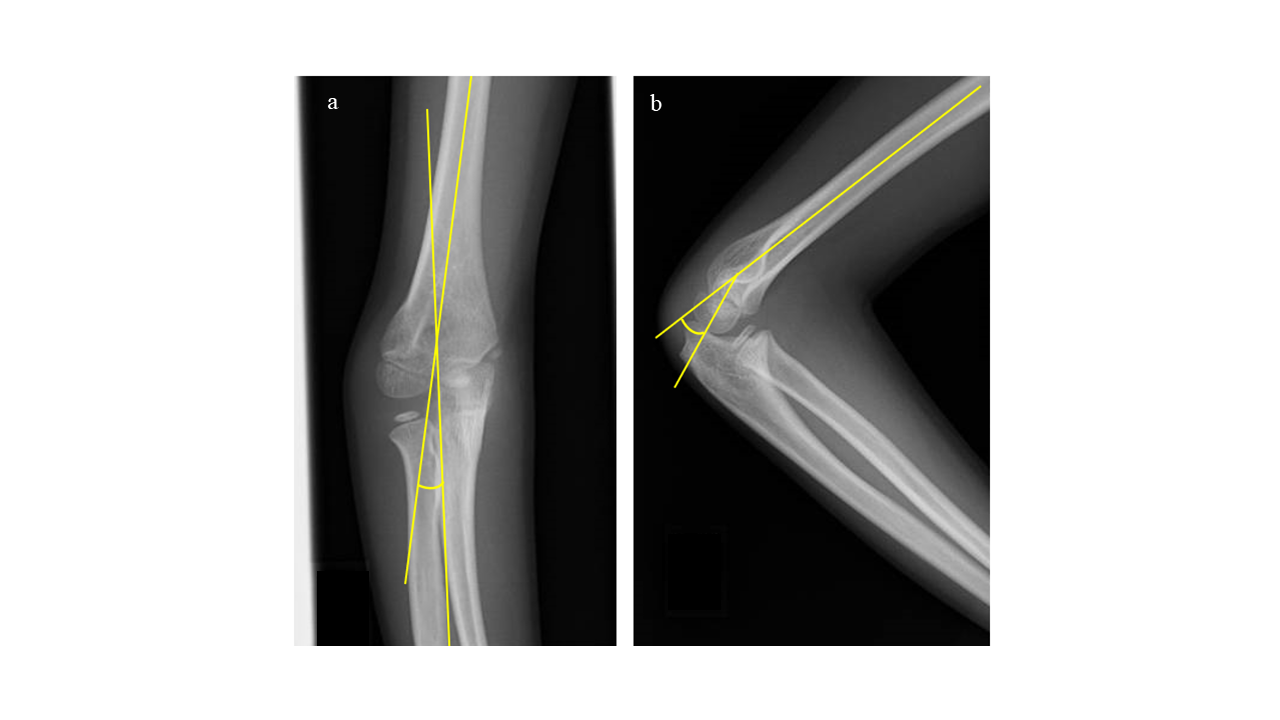


**Supplementary Fig. 1** Radiographic examination: measurement of HEW-A (a) and TA (b). TA, tilting angle; HEW-A, humerus-elbow-wrist angle

**
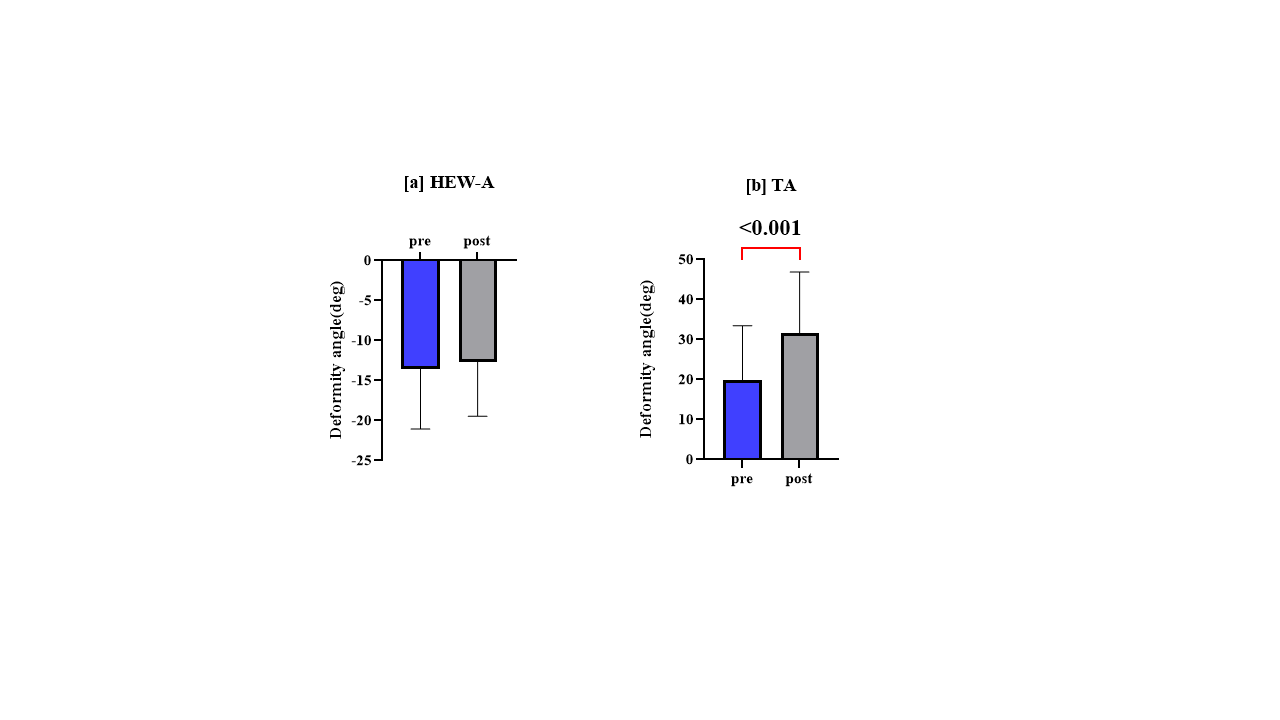
**

**Supplementary Fig. 2** Radiographic comparisons between bone union and CT evaluation. (a) HEW-A (b) TA. TA, tilting angle; HEW-A, humerus-elbow-wrist angle
