# Supplementary material for: Cubitus varus deformity following paediatric supracondylar humeral fracture remodelling predominantly in the sagittal direction: A three-dimensional analysis of eighty-six cases
Source: Int Orthop. 2024 May 10;48(8):2091–9. doi: 10.1007/s00264-024-06197-2 (PMC11246304; doi:10.1007/s00264-024-06197-2)
Supplement: Supplementary file 2 — Supplementary file2 (DOCX 28 KB) [file 264_2024_6197_MOESM2_ESM.docx]

**Title: Cubitus varus deformity following paediatric supracondylar humeral fracture remodelling predominantly in the sagittal direction: A three-dimensional analysis of eighty-six cases**

**Running Title: Remodelling capacity of cubitus varus deformity**

Tasuku Miyake, MD^a^ , Satoshi Miyamura, MD, PhD^a^ , Ryo Miki, MD^b^ , Ryoya Shiode, MD, PhD^a^ , Toru Iwahashi, MD, PhD^a^ , Arisa Kazui, MD^a^ , Natsuki Yamamoto, MD^a^ , Hiroyuki Tanaka, MD, PhD^a, c^ , Seiji Okada, MD, PhD^a^, Tsuyoshi Murase, MD, PhD^a, d^, Kunihiro Oka, MD, PhD^a, e^

^a^ Department of Orthopedic Surgery, Osaka University Graduate School of Medicine, Suita, Japan

^b^ Miki orthopedic surgery & internal medicine, Minoh, Japan

^c^ Department of Sports Medical Science, Osaka University Graduate School of Medicine, Suita, Japan

^d^ Department of Orthopedic Surgery, Bell Land General Hospital, Sakai, Japan

^e^ Department of Orthopedic Biomaterial Science, Osaka University Graduate School of Medicine, Suita, Japan

**Corresponding author:** Kunihiro Oka, MD, PhD

**Postal Address:** Department of Orthopedic Biomaterial Science, Osaka University Graduate School of Medicine, 2-2 Yamadaoka, Suita, Osaka 565-0871, Japan

**Email address:** [oka-kunihiro@ort.med.osaka-u.ac.jp](mailto:oka-kunihiro@ort.med.osaka-u.ac.jp)

**Phone:** +81-6-6879-3552; **Fax:** +81-6-6879-3559

**ORCID:**

Tasuku Miyake: 0009-0001-7404-2460

Kunihiro Oka: 0000-0002-7770-4634

**Association of patient-related outcome measures with functional prognosis and chief complaint**

We measured the range of motion as a clinical examination and used the Disabilities of the Arm, Shoulder and Hand (DASH) score as an elbow scoring system. We obtained DASH scores at CT scan for 17 patients. The mean range of motion of the elbow on the normal side of the patients responding to the DASH score was 150°, whereas the mean range of motion on the affected side was 140°. The mean DASH score tended to be low at 2.5 points. In other words, considering the results of the clinical examination and the DASH score, cosmetic disfigurement is often a problem in cubitus varus deformity in the early post deformity period (Supplementary Table2).

**Supplementary Table 2.** Patient data.

CT, computed tomography; SD, standard deviation; DASH, Disabilities of the Arm, Shoulder and Hand
