# Supplementary material for: Cubitus varus deformity following paediatric supracondylar humeral fracture remodelling predominantly in the sagittal direction: A three-dimensional analysis of eighty-six cases
Source: Int Orthop. 2024 May 10;48(8):2091–9. doi: 10.1007/s00264-024-06197-2 (PMC11246304; doi:10.1007/s00264-024-06197-2)
Supplement: Supplementary file 3 — Supplementary file3 (DOCX 22 KB) [file 264_2024_6197_MOESM3_ESM.docx]

**Conflict of Interest**

We wish to draw the attention of the Editor to the following facts that may be considered as potential conflicts of interest and the significant financial contributions to this work.

**Kunihiro Oka** has received funding from Japan Society for the Promotion of Science (JSPS) KAKENHI Grant No. JP 23K08612. Tsuyoshi Murase has received funding from Japan Society for the Promotion of Science (JSPS) KAKENHI Grant No. JP 22H03199. This source was involved in data collection, data analysis, and editing of the manuscript. Their immediate family has not received any financial payments or other benefits from any commercial entity related to the subject of this article.

**Tasuku Miyake,** their immediate family, and any research foundation with which they are affiliated did not receive any financial payments or other benefits from any commercial entity related to the subject of this article.

Satoshi Miyamura, their immediate family, and any research foundation with which they are affiliated did not

receive any financial payments or other benefits from any commercial entity related to the subject of this article.

**Ryo Miki,** their immediate family, and any research foundation with which they are affiliated did not receive any financial payments or other benefits from any commercial entity related to the subject of this article.

Ryoya Shiode, their immediate family, and any research foundation with which they are affiliated did not

receive any financial payments or other benefits from any commercial entity related to the subject of this article.

**Toru Iwahashi,** their immediate family, and any research foundation with which they are affiliated did not receive any financial payments or other benefits from any commercial entity related to the subject of this article.

**Arisa Kazui**, their immediate family, and any research foundation with which they are affiliated did not receive any financial payments or other benefits from any commercial entity related to the subject of this article.

**Natsuki Yamamoto,** their immediate family, and any research foundation with which they are affiliated did not

receive any financial payments or other benefits from any commercial entity related to the subject of this article.

**Hiroyuki Tanaka,** their immediate family, and any research foundation with which they are affiliated did not receive any financial payments or other benefits from any commercial entity related to the subject of this article.

**Seiji Okada,** their immediate family, and any research foundation with which they are affiliated did not receive any financial payments or other benefits from any commercial entity related to the subject of this article.

**Tsuyoshi Murase,** their immediate family, and any research foundation with which they are affiliated did not receive any financial payments or other benefits from any commercial entity related to the subject of this article.

**Kunihiro Oka,** their immediate family, and any research foundation with which they are affiliated did not receive any financial payments or other benefits from any commercial entity related to the subject of this article.
